# Supplementary material for: Testing gene-environment interactions for rare and/or common variants in sequencing association studies
Source: PLoS One. 2020 Mar 10;15(3):e0229217. doi: 10.1371/journal.pone.0229217 (PMC7064198; doi:10.1371/journal.pone.0229217)
Supplement: S1 Table — (PDF) [file pone.0229217.s001.pdf]

Supplementary Table 1: Summary results of association analysis for $TGFB1$ based on the COPD dataset. The p-values are shown for testing the gene's main effect (top panel), gene-by-smoking interaction with main effect (middle panel), gene-by-smoking interaction without main effect (bottom panel).

Gene's main effect					
trait	TOW	SKAT	WSS	CMC	VW-TOW
GasTrap	0.5593	0.9003	0.5339	0.8352	0.4900
ExacerFreq	**0.0217**	**0.014**	0.1853	0.4114	**0.0381**
Emph	0.758	0.7047	0.8966	0.5286	0.7236
Pi10	0.664	0.7048	0.9238	0.4610	0.5739
EmphDist	0.1455	0.5987	0.4047	0.3144	*0.0598*
6MWD	0.4022	0.5571	0.5668	0.9478	0.4620
FEV1	0.7251	0.6479	0.3214	0.8583	0.8054
COPD	0.5433	0.6646	0.8949	0.8915	0.466
Gene-by-smoking interaction with main effect					
trait	TOW-GE	ISKAT	WSS	CMC	VW-TOW-GE
GasTrap	0.1578	0.1137	0.1478	0.8198	0.1124
ExacerFreq	0.5955	0.4358	0.6358	0.8278	0.7039
Emph	0.1673	0.3471	0.4898	0.5697	0.2830
Pi10	0.4568	0.7892	0.2376	0.9567	0.2667
EmphDist	0.3946	0.195	0.6873	0.7030	0.4618
6MWD	0.4353	0.323	0.7365	0.8453	0.5958
FEV1	0.3821	0.3028	0.9218	0.8557	0.4720
COPD	0.7322	0.3162	*0.0842*	0.7964	0.763
Gene-by-smoking interaction without main effect					
trait	TOW-GE	ISKAT	WSS	CMC	VW-TOW-GE
GasTrap	0.5390	0.5870	0.9365	0.7568	0.7342
ExacerFreq	**0.0152**	**0.0114**	0.1846	0.1931	**0.0229**
Emph	0.5815	0.4886	0.8923	0.1611	0.6503
Pi10	0.3333	0.7769	0.6657	0.5945	0.1261
EmphDist	0.113	0.3230	0.6001	0.1137	*0.0554*
6MWD	0.2909	0.2056	0.5110	0.9647	0.4708
FEV1	0.9639	0.9736	0.3431	0.9034	0.9849
COPD	0.4066	0.2779	0.5472	0.3532	0.5118

Note: The bold numbers represent p-values of significant tests (significance level = 0.05); the italic numbers represent p-values between 0.05 and 0.1.
