# Supplementary material for: Testing gene-environment interactions for rare and/or common variants in sequencing association studies
Source: PLoS One. 2020 Mar 10;15(3):e0229217. doi: 10.1371/journal.pone.0229217 (PMC7064198; doi:10.1371/journal.pone.0229217)
Supplement: S2 Table — (PDF) [file pone.0229217.s002.pdf]

Supplementary Table 2: Summary results of association analysis for TNF based on the COPD dataset. The p-values are shown for testing the gene's main effect (top panel), gene-by-smoking interaction with main effect (middle panel), gene-by-smoking interaction without main effect (bottom panel).

Gene's main effect					
trait	TOW	SKAT	WSS	CMC	VW-TOW
GasTrap	0.7673	0.9765	0.7969	0.7312	0.7717
ExacerFreq	0.652	0.5594	0.8313	0.9452	0.7211
Emph	0.1706	0.7850	0.1342	0.3026	0.1530
Pi10	0.9175	0.9963	0.6261	0.8963	0.9364
EmphDist	**0.0404**	**0.0281**	**0.0067**	*0.0733*	*0.0591*
6MWD	0.5841	0.6964	0.7973	0.8775	0.6520
FEV1	0.1639	0.1464	0.7166	0.4536	0.1979
COPD	0.2552	0.7312	0.6918	0.5454	0.3298
Gene-by-smoking interaction with main effect					
trait	TOW-GE	ISKAT	WSS	CMC	VW-TOW-GE
GasTrap	0.8486	0.7514	0.8429	**0.0171**	0.6855
ExacerFreq	0.1008	*0.0905*	**0.0129**	*0.0767*	*0.0944*
Emph	0.6671	0.4443	0.6205	**0.0035**	0.4611
Pi10	0.153	0.1138	**0.0478**	0.1286	0.1903
EmphDist	0.9668	0.6187	0.9004	0.1371	0.9032
6MWD	0.7344	0.7057	0.2871	0.3242	0.4373
FEV1	0.6631	0.7299	0.3483	**0.0189**	0.4102
COPD	0.7021	0.2554	0.2713	**0.0021**	0.5111
Gene-by-smoking interaction without main effect					
trait	TOW-GE	ISKAT	WSS	CMC	VW-TOW-GE
GasTrap	0.6367	0.8787	0.8590	0.1255	0.763
ExacerFreq	0.7499	0.6724	0.3429	0.6911	0.8292
Emph	0.1442	0.2904	0.1122	**0.007**	0.1871
Pi10	0.6773	0.7254	0.5744	0.7066	0.743
EmphDist	*0.0881*	**0.031**	**0.0135**	**0.0156**	0.1155
6MWD	0.8877	0.734	0.8354	0.4127	0.9174
FEV1	0.3381	0.2001	0.9849	0.1204	0.4876
COPD	0.5218	0.357	0.9124	0.3026	0.5752

Note: The bold numbers represent p-values of significant tests (significance level = 0.05); the italic numbers represent p-values between 0.05 and 0.1.
