# Supplementary material for: Testing gene-environment interactions for rare and/or common variants in sequencing association studies
Source: PLoS One. 2020 Mar 10;15(3):e0229217. doi: 10.1371/journal.pone.0229217 (PMC7064198; doi:10.1371/journal.pone.0229217)
Supplement: S3 Table — (PDF) [file pone.0229217.s003.pdf]

Supplementary Table 3: Summary results of association analysis for *SFTPB* based on the COPD dataset. The p-values are shown for testing the gene's main effect (top panel), gene-by-smoking interaction with main effect (middle panel), gene-by-smoking interaction without main effect (bottom panel).

Gene's main effect					
trait	TOW	SKAT	WSS	CMC	VW-TOW
GasTrap	0.1279	0.4772	0.9110	0.5191	0.2171
ExacerFreq	0.24	0.1329	0.7890	0.5492	0.3884
Emph	0.2214	0.4136	0.5173	0.5745	0.2406
Pi10	0.4126	0.1985	0.9056	0.4445	0.3200
EmphDist	0.1613	0.4338	**0.0400**	0.3474	0.2690
6MWD	0.2910	0.9067	0.1191	0.5159	0.2451
FEV1	0.4759	0.7640	0.4265	0.8644	0.4601
COPD	0.2586	0.5401	0.7786	0.7771	0.2291
**Gene-by-smoking interaction with main effect**					
trait	TOW-GE	ISKAT	WSS	CMC	VW-TOW-GE
GasTrap	0.1830	0.2509	0.2345	0.1483	0.2407
ExacerFreq	0.3999	0.9885	0.9726	0.6179	0.5396
Emph	0.5708	0.354	0.5731	0.2823	0.6861
Pi10	0.2952	0.9434	0.5964	0.9452	0.4632
EmphDist	0.4199	0.3046	0.2216	0.5887	0.4675
6MWD	0.4975	0.8315	0.4853	0.9314	0.4343
FEV1	0.4028	0.2783	0.7065	0.3780	0.4717
COPD	0.2027	**0.0299**	0.6218	0.3594	*0.0921*
**Gene-by-smoking interaction without main effect**					
trait	TOW-GE	ISKAT	WSS	CMC	VW-TOW-GE
GasTrap	*0.0956*	0.6685	0.3520	0.4041	0.1367
ExacerFreq	*0.096*	0.258	0.9710	0.3019	0.1577
Emph	0.2444	0.3336	0.8869	0.3291	0.2845
Pi10	0.1251	0.2643	0.7202	0.6857	0.1398
EmphDist	**0.0488**	**0.013**	**0.0415**	0.1282	0.1068
6MWD	0.1800	0.9499	0.1137	0.8019	0.1656
FEV1	0.6488	0.972	0.6629	0.9916	0.6495
COPD	0.284	0.1437	0.6085	0.4089	0.4708

Note: The bold numbers represent p-values of significant tests (significance level = 0.05); the italic numbers represent p-values between 0.05 and 0.1.
