# Supplementary material for: Testing gene-environment interactions for rare and/or common variants in sequencing association studies
Source: PLoS One. 2020 Mar 10;15(3):e0229217. doi: 10.1371/journal.pone.0229217 (PMC7064198; doi:10.1371/journal.pone.0229217)
Supplement: S4 Table — (PDF) [file pone.0229217.s004.pdf]

Supplementary Table 4: Summary results of association analysis for $EPHX1$ based on the COPD dataset. The p-values are shown for testing the gene's main effect (top panel), gene-by-smoking interaction with main effect (middle panel), gene-by-smoking interaction without main effect (bottom panel).

Gene's main effect					
trait	TOW	SKAT	WSS	CMC	VW-TOW
GasTrap	0.7728	0.2626	0.7731	0.9697	0.4303
ExacerFreq	0.6128	0.7044	0.3495	0.9635	0.5598
Emph	0.8557	0.4199	0.9558	0.991	0.6585
Pi10	0.3839	0.3405	0.7855	0.3312	0.446
EmphDist	0.1687	0.1112	0.4459	0.4629	0.1205
6MWD	0.6754	0.8188	0.6769	0.9548	0.4413
FEV1	0.9531	0.9048	0.8531	0.8322	0.9706
COPD	0.7939	0.6854	0.716	0.5841	0.8423
**Gene-by-smoking interaction with main effect**					
trait	TOW-GE	ISKAT	WSS	CMC	VW-TOW-GE
GasTrap	0.1018	0.1247	**0.0424**	0.8863	0.1281
ExacerFreq	0.2729	0.1552	0.1031	0.5298	0.4284
Emph	*0.0992*	0.2882	*0.0681*	0.8969	*0.0843*
Pi10	0.6705	0.87	0.7482	0.3963	0.6855
EmphDist	0.6352	0.7354	0.3184	0.9652	0.4601
6MWD	0.5196	0.3792	0.9083	0.6618	0.4866
FEV1	*0.0804*	0.1883	**0.0446**	0.9047	0.1041
COPD	0.5928	0.2741	0.2904	0.5814	0.4408
**Gene-by-smoking interaction without main effect**					
trait	TOW-GE	ISKAT	WSS	CMC	VW-TOW-GE
GasTrap	0.4761	0.1824	0.465	0.6511	0.4959
ExacerFreq	0.8809	0.9565	0.8243	0.8953	0.7906
Emph	0.2604	0.1551	0.3715	0.7569	0.3959
Pi10	0.1522	0.1918	0.9936	*0.0581*	0.2379
EmphDist	0.1325	0.1106	0.9444	0.3218	0.1581
6MWD	0.7143	0.7297	0.5812	0.971	0.6397
FEV1	0.4482	0.2695	0.4726	0.683	0.4983
COPD	0.5561	0.3064	0.3414	0.3122	0.5136

Note: The bold numbers represent p-values of significant tests (significance level = 0.05); the italic numbers represent p-values between 0.05 and 0.1.
