# Supplementary material for: Testing gene-environment interactions for rare and/or common variants in sequencing association studies
Source: PLoS One. 2020 Mar 10;15(3):e0229217. doi: 10.1371/journal.pone.0229217 (PMC7064198; doi:10.1371/journal.pone.0229217)
Supplement: Attachment — Submitted filename: Response to Reviewers.docx [file pone.0229217.s006.docx]

Heming Wang, PhD
Academic Editor
PLOS ONE

Re: Manuscript Number: PONE-D-19-27761R1, title “Testing gene-environment interactions for rare and/or common variants in sequencing association studies”

Dear Dr. Wang,

We thank you for your encouraging evaluation for our manuscript “Testing gene-environment interactions for rare and/or common variants in sequencing association studies” (Manuscript Number: PONE-D-19-27761R1). We appreciate the editor's patient work in organizing the reviewing process. After thoroughly and carefully addressing the reviewers' comments and making corresponding revisions in the manuscript highlighted in red, we are now resubmitting the paper for your further consideration. Please find our point-by-point response to the reviewers' comments in the letter below.

Thank you for the helpful and constructive review. We feel that the manuscript is much improved and hope you now find it suitable for publication in PLOS ONE.

Han Hao

Assistant Professor of Statistics

Department of Mathematics

University of North Texas

1155 Union Circle Denton, TX 76201.

Email: [Han.Hao@unt.edu](mailto:Han.Hao@unt.edu)

**Response to reviewers' comments:**

***Reviewer #1:***

*In this manuscript, authors proposed methods to detect gene-environment (GE) interactions for rare and/or common variants. A generalized linear model was developed and a score test was established for testing GE interactions. Permutation tests were used for obtaining p-values. Simulation studies were conducted to validate the properties of the test. It was showed that the proposed test was more powerful than existing ones in this area. The application of proposed model has been demonstrated with an empirical analysis of data for Chronic obstructive pulmonary disease. It is a nice work and provides biomedical researchers with useful methodology to address scientific questions related to GE interactions, particularly of rare variants.*

**Reply:** We thank the reviewer for the very positive comments.

*My questions are about the computation.

1. I understand permutation tests have high computational cost when sample size is large and/or the number of features/genes is large. It was mentioned that HPC infrastructure had been utilized for this study. I wonder if it is feasible to perform the proposed test on a personal computer as it is more convenient for average users.*

**Reply:** Yes. The reviewer is correct. The permutation tests have high computational cost. We added a paragraph in the Discussion section to discuss the computation time and a possible way to make an improvement.

The proposed test statistic TOW-GE does not have an asymptotic distribution and a permutation procedure is needed to estimate its P-value, which is time consuming compared to methods with asymptotic distributions. To save time when applying the proposed methods in genetic association studies, we can use the “step-up" procedure (Barnett, 2017; Pan, 2014) to determine the number of permutations, which can show evidence of association based on a small number of permutations first (e.g.1,000) and then a large number of permutations are used to test the selected potentially significant genes. Specifically, the computation time of P-value estimation of TOW-GE and VW-TOW-GE for a gene in the real data analysis was about 30 seconds using our R program on 6 Dell PowerEdge C6320 servers. Each server has two 2.4GHz Intel Xeon E5-2680 v4 fourteen-core processors and 600 MB average memory. We have uploaded the R program on GitHub at  [https://github.com/Jianjun-CN/Single-GE](https://nam04.safelinks.protection.outlook.com/?url=https%3A%2F%2Fgithub.com%2FJianjun-CN%2FSingle-GE&data=02%7C01%7CXuexia.Wang%40unt.edu%7Ca18eb96b80cc4725149908d785bae276%7C70de199207c6480fa318a1afcba03983%7C0%7C0%7C637124910017039566&sdata=uLYqc6fkdr%2BMgtZE7mjLYybCP9Tr3Pv6e%2FPAKNz%2FqLg%3D&reserved=0).

*2. Are the code of your model/test available to public?*

**Reply:** Yes. We have uploaded the R program onto GitHub at  [https://github.com/Jianjun-CN/Single-GE](https://nam04.safelinks.protection.outlook.com/?url=https%3A%2F%2Fgithub.com%2FJianjun-CN%2FSingle-GE&data=02%7C01%7CXuexia.Wang%40unt.edu%7Ca18eb96b80cc4725149908d785bae276%7C70de199207c6480fa318a1afcba03983%7C0%7C0%7C637124910017039566&sdata=uLYqc6fkdr%2BMgtZE7mjLYybCP9Tr3Pv6e%2FPAKNz%2FqLg%3D&reserved=0).

**Reviewer #2:**

*In this paper, Zhao and colleagues describe a novel method for rare and common variants gene-environment interaction testing in sequencing association studies. The method builds upon substantial existing work and is more powerful than existing methods by using an adaptive weight selection procedure. The text overall is very clearly written.*

**Reply:** We thank the reviewer for the very positive comments.

*However, I do have some minor concerns that I would like the authors to address.
(1) The permutation procedure for the p-value calculation is time-consuming. Barnett et.al (JASA, 2017) provided a scheme for permutation test to save computation time. It would be good to incorporate it in the paper.*

**Reply:** We thank the reviewer for the suggestion. We added a paragraph in the Discussion section to discuss the computation time and a possible way to make an improvement based on the scheme for a permutation test provided by Barnett et.al (JASA, 2017).

*(2) In the real data application, the authors only test for association of two known genes. It would be great for the authors to show the results of the gene-based analysis of the whole genome.*

**Reply:** The data of the COPDGene Study that we used in the real data analysis is sufficiently large and appropriately designed for analysis of COPD, which consists of more than 5,000 non-Hispanic Whites (NHW) participants genotyped using the Illumina OmniExpress platform. The genotype data is a genome-wide association study data mainly focusing on testing for traits or COPD associated common genetic variants. To test gene-environment interactions for both rare and common genetic variants, we need to impute the COPD genotype data using the EUR haplotypes from the 1000 Genome Project as references. It is very time consuming to impute genotypes for SNPs on all of the 22 chromosomes. To provide sufficient assessment for the proposed methods and save time, we performed analysis for four more genes reported in the literature. Please see page 7 in the main manuscript and the results in the supplemental materials.

**References:**

Barnett I, Mukherjee R, and Lin X. The generalized higher criticism for testing SNP-set effects in genetic association studies. Journal of the American Statistical Association. 2017; 112(517):64-76.

Pan W, Kim, J, Zhang Y, Shen X, Wei P. A powerful and adaptive association test for rare variants. Genetics. 2014; 197(4):1081-1095.
